# The effects of the COVID-19 pandemic on the treatment of allergic eye diseases

**DOI:** 10.1097/ACI.0000000000000766

**Published:** 2021-07-15

**Authors:** Andrea Leonardi, Elena Salami, Oren Mark Feuerman, Fabiano Cavarzeran

**Affiliations:** a Department of Neuroscience, Ophthalmology Unit, University of Padua, Padua; b Department of Ophthalmology, Santa Maria Della Misericordia Hospital, Rovigo, Italy

**Keywords:** COVID-19, management recommendations, ocular allergy, vernal keratoconjunctivitis

## Abstract

To analyze if recommendations given before and during the COVID-19 pandemic are still a valid option for patients suffering of ocular allergy (OA).

**Recent findings:**

During the last year many doctors and patients requested suggestions for the treatment of OA patients in COVID-19 time. Most of them were given by phone calls and emails following the recommendations given by Scientific Societies.

**Summary:**

Considering the current multiple problems related to the COVID-19 pandemic, OA has not been considered a priority, even though patients need treatments. Topical antiallergic drugs are still the first option to treat all kind of OA, with the addition of topical corticosteroids in the severe forms of vernal and atopic keratoconjunctivitis (VKC and AKC) even in patients at risk of COVID-19. Topical immunomodulation is still recommended in severe forms of VKC and AKC unless the patient is infected. The number of patients treated with these drugs in our Center was similar than previous years. The risk to have a VKC patient affected by COVID-19 is similar to the general pediatric population but with a lower OR. In 2021, still in COVID-time, the management of OA should follow the previous recommendations with an update due to the risk of infection.

## INTRODUCTION

The severe acute respiratory syndrome coronavirus-2 (SARS-CoV-2) knows as COVID-19 pandemic posed significant challenges to medical practice and clinical care. The virus is transmittable from asymptomatic, presymptomatic, and symptomatic positive patients, by contact, droplets of saliva, nasal and oral discharge and fomites. Furthermore, the airborne transmission increases the risk of infection for healthcare workers, who routinely visits patients at close distance [1].

Italy was the first European country to be interested in the pandemic. This happened in proximity of the spring and the majority of allergic patients were confined to their homes due to the lockdown. After two months, restrictions were reduced and people were allowed to take walks, go jogging, and return to work. In the spring/summer, patients affected by ocular allergy (OA) became symptomatic even when wearing a mask since the eyes remain unprotected. Patients with OA were unable to access their usual specialist centers to receive periodic treatments because of the lockdown. Consequently, there was a potential risk for the patients of stopping or modifying their treatment. In case of complications, assessment in the emergency service would have exposed the patients to the potential risk of COVID-19 infection.

Ophthalmologists visit patients at relatively short distance to the face with hands at direct contact with tears and in a very proximity to the patient's nose and mouth [2]. To support the high risk during routine slit lamp examination, it can be cited the Li Wenliang case, the ophthalmologist from Wuhan, who first raised the alarm about the novel coronavirus and later died of the disease (https://www.aao.org/headline/coronavirus-kills-chinese-whistleblower-ophthalmol).

The conjunctiva is a potential gateway for the SARS-CoV-2 to enter the body especially when working at a close distance to the patient's airways [3]. The slit lamp examination is preferably done in dark or dim light rooms, with blinds, windows and doors closed, creating a poorly ventilated setting [4,5^▪^]. We and others reported that SARS-CoV-2 receptors are expressed by both conjunctiva and cornea [6^▪^,^7^,8^▪^] and that angiotensing converting enzyme2 is overexpressed in diseased conjunctival tissue [9] confirming the hypothesis that the ocular surface may represent an entry point for the virus. The prevalence of conjunctivitis has been reported from 0.8 to 7.9% of COVID-19 patients manifesting as conjunctival redness, irritation, foreign body sensation, epiphora and chemosis. These symptoms are more common in severe COVID-19 patients, but conjunctivitis may be the only manifestation of the disease. It has been reported that SARS-CoV-2 was present on the ocular surface in 57% of COVID-19 patients [10] and detected also in patients with negative nasopharyngeal swab. However, the infectivity of the virus detected on the ocular surface remains controversial [11]. 

**Box 1:**
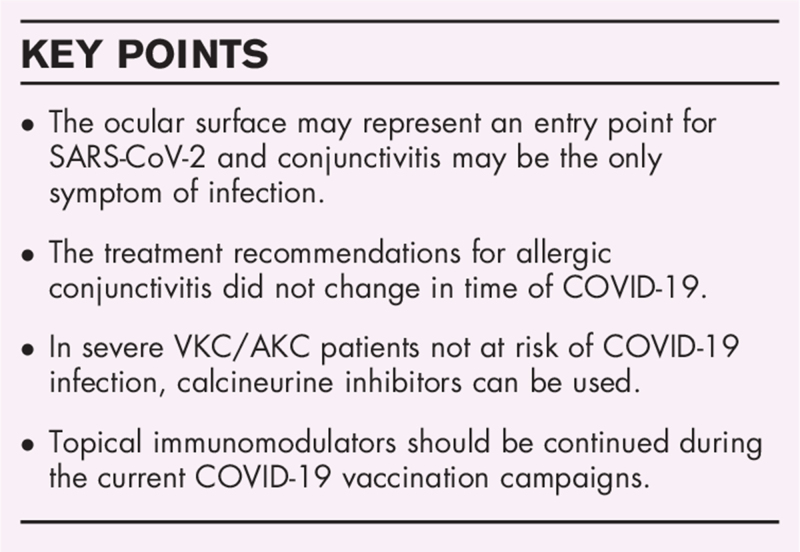
no caption available

## OCULAR ALLERGY

The term OA refers to a collection of ocular surface disorders that affect the eyelid, conjunctiva and cornea. Diagnosis and management of OA is a common practice for allergists, pediatricians, rhinologists, ophthalmologists and general practitioners. They are used to manage the most common IgE-mediated forms of OA, intermittent/seasonal and persistent/perennial allergic conjunctivitis (SAC and PAC) [12], whereas vernal and atopic keratoconjunctivitis (VKC and AKC) should be managed by specialized centers [12]. Several ocular disorders, such as dry eye, blepharitis, infections, toxic and mechanical conjunctivitis, have clinical manifestations similar to OA but may be difficult to distinguish from OA without an evaluation at the slit lamp [13]. To date, there is no specific clinical and laboratory test suitable for the diagnosis and monitoring of OA. Medical history, signs and symptoms, laboratory tests, and imaging techniques can be useful [13,14^▪▪^,^15^]. The management of severe forms of VKC and AKC, which can result in corneal complications and cause sight threatening sequelae, requires specialized expertise [12,14^▪▪^,^16^^▪▪^]. All these procedures became difficult during COVID-19 pandemic.

## METHODS

A literature review was performed in PubMed, using the following Keywords: Allergic Conjunctivitis or Ocular Allergy [AND] COVID19, SARS-CoV-2, coronavirus pandemic. A review of European and International guidelines on the management of allergic patients during the COVID-19 pandemic has been performed. Manual search of the reference lists of selected studies was done and relevant studies identified. No time or language limitations were established. Papers were selected according to the information provided on the title and abstract. Each topic was reviewed and analyzed by all Authors.

## COVID-19 RECOMMENDATIONS FOR THE PATIENT CARE WITH ALLERGIES

In 2019 the European Academy of Allergy and Clinical Immunology (EAACI) working group on OA (WG-OA) have published recommendations on the management of OA [16^▪▪^]. Considering the numerous problems caused by the COVID-19 pandemic, including the health system crisis and limited access to consultants, it was reasonable to verify whether these recommendations were still applicable. Scientific Societies have expressed recommendations concerning the assessment of allergic conditions during the COVID-19 pandemic as results of experts’ panel opinion, however, without focusing on OA. The recommendations for the management of allergy and asthma have been updated following the COVID-19 pandemic, again without mentioning OA. A recent position paper provides, in nine sections, relevant aspects for the care of allergic patients to maintain high standards in the daily clinical care of patients ensuring necessary safety [17^▪▪^]. The use of telemedicine is considered a useful tool for providing medical advice when physical presence is impossible or limited. However, limitations of this technology are the lack of objective data from physical examination, data security and protection. Although preventive measures for allergic symptoms during the COVID-19 pandemic are similar in most of the allergic diseases, different recommendations may be provided for patients with suspected SARS-CoV-2 infection or diagnosed COVID-19 disease versus noninfected individuals or patients who have recovered from COVID-19 infection. After recovery, standard allergy care should be resumed with prior interdisciplinary consultation, before any further diagnostic or therapeutic procedure [17^▪▪^]. Clinical trials should be continued maintaining all the anti-COVID 19 safety measures [17^▪▪^]. Another expert panel consensus document from the USA and Canada offered a prioritization rational to help guide decision-making in COVID-19 when to limit patient access during the COVID-19 pandemic [18^▪^].

All these recommendations are aimed at reducing unnecessary physical interactions. Implementation of telemedicine and deletion of elective procedures have been encouraged by guidelines to prevent the spread of the virus by providing social distance measures. The role of telemedicine in ophthalmology has become almost a regular practice in the last year through tele-consultation, visioning pictures and video send directly by patients or by other doctors, however, telehealth capacities remain limited in terms of accurate assessment of ocular surface diseases. A survey of 1260 Indian ophthalmologists showed that 77.5% of them began telephonic or video consultations with their patients since lockdown onset [19]. Despite all the enthusiasm, tele-screening as currently performed with the available tools is still not capable of completely replacing a standard ophthalmic examination for the assessment of ocular surface diseases including OA. Although waiting for new emerging technologies and future implementation of imaging modalities and artificial intelligence, decision-making algorithms can help eye-practitioners remotely screen their patients to assess the optimal time for follow-up appointments [20^▪^].

## MANAGEMENT OF OCULAR ALLERGY IN COVID-19 TIME

Independently from the phenotype of OA, treatment should follow a stepwise approach based on clinical sign and symptoms severity, using topical antihistamines, mast cell stabilizers, or dual-action drugs as the first choice of treatment with the addition of brief courses of topical corticosteroids in the severe forms or long-term use of topical immunomodulators [14^▪▪^,^16^^▪▪^].

Topical corticosteroids and immunomodulators have the potential to increase local susceptibility, persistence and reactivation of viral infection. Although the presence of SARS-CoV-2 in tears has rarely been detected in infected individuals [21,22], conjunctivitis may be a sign of COVID-19, prior to or after the onset of respiratory symptoms [23].

Socio-psychological mechanisms play an important role in the development, exacerbation, and perception of symptoms in allergic patients. The general population is highly sensitive to people exhibiting ocular and respiratory symptoms during the COVID-19 pandemic. This increases the risk of stigmatization of patients with allergies increasing further patients’ psychosocial stress. Thus, optimal medical and psychological care for patients with allergies, including OA, during the COVID-19 pandemic is essential [17^▪▪^].

The impact of quarantine on the quality of life, daily activities, psychological health and the recurrence of allergic conjunctivitis were moderate [24]. Males were not greatly affected emotionally by the flare-up of eye symptoms during quarantine [24], whereas a previous study reported that males were more bothered by eye symptoms [25]. Patients were less likely to have allergy symptoms and disease exacerbations, because of the less outdoor allergen exposure. Similarly, during the lockdown, there was a decrease trend of allergic rhinitis (AR) symptoms due to the increased indoor activities [26]. Interestingly, a study conducted in nurses with AR working with COVID-19 patients, revealed that the use of face mask reduced symptom severity in chronically affected AR subjects [27] confirming that face mask use reduces allergic reactions.

In the early spring 2020, at the beginning of COVID-19 pandemic, a survey was conducted by members of the EAACI-OA Working Group, regarding the management of clinical forms of OA in the time of COVID-19 [28^▪▪^]. The panel divided OA patients into different groups defined by different situations: a) *not at risk* of SARS-Cov-2 infection; b) *at risk* of SARS-Cov-2 infection; c) *current* SARS-Cov-2 infection; d) *previous* SARS-Cov-2 infection. Treatment recommendations were evaluated to confirm agreement with previously published recommendations [16^▪▪^]. The panel opinion confirmed that the treatment recommendations for SAC are still valid despite COVID-19-related problems. Eye rubbing is not recommended in any type of OA, especially in time of COVID-19 and it is essential to prevent this behavior. Topical antiallergic eyedrops were confirmed to be the first choice of treatment (Table 1). The use of only systemic antihistamines or low-dose corticosteroids in case of no response was not accepted as a valid option. Regarding VKC/AKC, the combination of topical antiallergic drugs remains definitively the first-line treatment, with the addition of topical corticosteroids as pulse therapy, as second-line in case of exacerbations (Table 1). There was a disagreement on the use of topical corticosteroids in patients with current COVID-19 infection. The use of low dose of corticosteroids on the long term is not recommended independently from the current pandemic. If there is a clinical need to use corticosteroids for long time, topical immunomodulators should be considered. In severe VKC/AKC patients not at risk of COVID-19 infection these drugs can be used. In case a patient is at risk, with current or previous COVID-19 infection, immunomodulators should not be started. In our opinion, topical calcineurine inhibitors in severe VKC at risk should not be stopped if already ongoing, whereas in patients with previous COVID-19 infection, the decision should be taken individually. Not using topical immunomodulators may obligate an excessive use of corticosteroids leading to potential sight-threatening complications. It should be remarked that there is a negligible or a very low systemic absorption of topical cyclosporine [29].

**Table 1:** Treatment options for ocular allergy in COVID-19 time

SAC	Topical antiallergic drops	Low dose topical corticosteroids (if no response)	Only oral antihistamines
SARS-CoV-2 infection			
Not at-risk	YES	NO	NO
At risk	YES	NO	NO
Current infection	YES	NO	NO
Previous infection,	YES	NO	NO

VKC/AKC	Topical antiallergic drops	Topical corticosteroids as needed as pulse therapy	Topical calcineurin inhibitors
SARS-CoV-2 infection			
Not at-risk	YES	YES	Y/N
At risk	YES	Y/N	NO
Current infection	YES	NO	NO
Previous infection	YES	Y/N	NO

AKC, atopic keratoconjunctivitis; *N*, agreement < 50%; SARS-CoV-2, severe acute respiratory syndrome coronavirus-2; VKC, vernal keratoconjunctivitis; Y/N, agreement between 50 and 75%; YES, agreement between expert > 75%.

The use of systemic biologicals is a relatively new option for the management of refractory AKC. It is not recommend starting immunosuppression in patients with current SARS-CoV-2 infection as it is considered a controversial treatment strategy (Table 2). In patients at risk, local treatment is preferred. The addition of systemic immunomodulation treatment was not considered a good choice for patients at-risk whereas or with previous infection. Recent recommendations on the use of biologics in allergy, suggest that in patients with nonsuspected or not-proven SARS-CoV-2 infection, the use of biologicals can be continued unchanged or can be re-started [30].

**Table 2:** Treatment options for systemic immunosuppression in refractory atopic keratoconjunctivitis (AKC)

Refractory AKC		Add systemic immunomodulation			
	Only topical treatment	Corticosteroids at high dose	Cyclosporine	Azathioprine	Dupilumab
SARS-Cov-2					
At-risk	Y/N	NO	NO	NO	NO
Previous infection	Y/N	NO	NO	NO	NO

*N*, agreement < 50%; SARS-CoV-2, severe acute respiratory syndrome coronavirus-2; Y/N, agreement between 50 and 75%; YES, agreement between expert > 75%.

Since vaccination campaigns have been started, patients with OA are not at increased risk for allergic reactions after COVID-19 vaccination. Topical immunomodulators should be continued during the current COVID-19 vaccination campaigns. However, the intervals of biological treatment may need to be slightly adjusted [31].

## 2020 EXPERIENCE WITH OCULAR ALLERGY PATIENTS IN COVID-TIME

During the first months of COVID-19 pandemic and lockdown, OA patients were visited in our Center only for emergencies. In-person consultation were performed accordingly to the general recommendations from the European Centre for Disease Control, the World Health Organization and the National, Regional and University Hospital guidelines. Physical contact with the patients was minimized and additional diagnostic tools such as tear sampling, cytology, Schirmer test, contact instrumental procedures (tonometry, confocal microscopy) were avoided. The majority of consultancies were operated by phone or E-Mail. Digital health solutions, especially the use of telemedicine, have been previously proposed as a useful tool to provide medical advice remotely [32]. Pictures taken by the patient or doctors using cellphones and sent to the ophthalmologist were particularly useful. The organization of our Ocular Immunology Service during the COVID-19 pandemic was in line with the recommendations suggested by International Societies [17^▪▪^,^33^]. Since June 2020, there were no further restrictions on consultancies, even though all preventing measures were maintained. In the springtime 2020, the majority of OA patients reported a late start of ocular symptoms or much less symptoms compared the previous seasons due to the less outdoors exposure. This was even more evident in severe OA, such as VKC. Once the restrictions in outdoors activities were reduced, patients started to be symptomatic as previous years. The summer 2020 was the hottest summer ever in our Region with high-temperature peaks alternating to summer storms, not the ideal weather for VKC patients. In fact, the number of VKC patients treated with cyclosporine or tacrolimus compounded preparations (110 patients) was similar to that of 2019 but the number of preparations done in the spring/summer was lower because of the later start of symptoms. So far, only one VKC children reported to have been affected by COVID-19 in the spring 2020, with diarrhea as the only symptom.

In Europe, the mean prevalence of VKC has been estimated 3.2/10.000 with lower prevalence in the Northern countries compared with the Mediterranean countries. In our Region, the prevalence of VKC was estimated 4/10.000 under 15 years of age [34], therefore higher than reported in the European study. These numbers are lower in most EU and non-EU countries, but higher in areas, such as India and Africa, where VKC is more prevalent [35,36]. Since it is still too early to have a real number of the co-morbidity between VKC and COVID-19, knowing that the prevalence of COVID-19 in pediatric population (0–14) in Padova great area is 6.4% (https://www.azero.veneto.it), knowing the prevalence of VKC in our area, the co-morbidity of asthma and VKC in our area (14.6%) and the COVID-19 prevalence in asthma population [37,38] we calculated that the odd ratio (OR) for VKC to be associated to COVID-19 is OR = 0.88 (95% CI, 0.66–1.16). Even though this is a calculation, the risk of encountering a VKC patient positive for SARS-CoV-2 is low with a tendency to be lower than the non-VKC subjects. Interestingly, it has been suggested that a Th2-skewed immunity may be protective against severe COVID-19 disease [39^▪^].

## CONCLUSION

The recent recommendations of for the management of OA [5^▪^] are still valid at the time of COVID-19 even after one year experience of repeated lockdowns and re-opening (Table 3). We highlight the lack of evidence-based literature regarding the management of OA during the COVID-19, however, recommendation given by the expert opinion in 2020 have been followed and well accepted by doctors and patients. In 2021 the management of OA should follow the previous recommendations [5^▪^,^28^^▪▪^]. Particular caution is still recommended for the use of topical corticosteroids and systemic immunomodulators since no definitive consensus has been obtained, particularly for patients with current or previous COVID-19 infection.

**Table 3:** Recommendations for ocular allergy treatment in the time of COVID-19 Modified from (Leonardi A, al. Allergy. 2019;74(9):1611–30.)

A	How to treat IgE-mediated diseases SAC and PAC	
1	Avoidance of clinically relevant allergens is the first step in the prevention of ocular allergy symptoms	YES
2	Topical antihistamines, mast cell stabilizers, or dual-action drugs are the first choice of treatment, and all effective in reducing signs and symptoms	YES
3	Dual-acting agents with combined mast cell stabilizer and antihistaminic function increase the possibility of symptom improvement and have a faster relief of symptoms compared to mast cell stabilizers	YES
4	Avoid topical corticosteroids, as they are rarely needed	Y/N
5	Intranasal corticosteroids are effective and well tolerated in the treatment of ocular symptoms associated with ARC, but should not be used if only ocular signs and symptoms are present	Y/N
6	Topical vasoconstrictors alleviate only hyperemia and should be used with caution for a short period of 5-7 days because of side effects and tachyphylaxis	YES
7	Systemic antihistamines should be used in acute forms or when ocular symptoms are associated with other allergic comorbidities	YES
8	Leukotriene inhibitors are reported to be less efficacious than oral antihistamines in adult SAC patients	YES
9	Consider SIT when specific sensitization is the main cause of ocular allergy, as it is effective for the treatment of ARC to seasonal allergens and perennial allergens	YES
10	SLIT has been shown to be effective in reducing total and individual ocular symptom score in subjects with conjunctivitis	YES

B	How to treat persistent/chronic forms (IgE- and non-IgE-mediated) VKC and AKC	
1	Avoidance of specific and nonspecific triggers is the first step in the prevention of ocular allergy symptoms	YES
2	Use cold compresses, good eyelid hygiene, and lubricants	YES
3	Topical antihistamines, mast cell stabilizers, or dual-action drugs are the first treatment choice and may be used in combination. They should be used frequently during the day and during the whole season	YES
4	Systemic antiallergic drugs should be used when ocular symptoms are associated with other allergic comorbidities	YES
5	Topical corticosteroids should be used as short, pulsed therapy, in acute exacerbations or when the cornea is involved, under ophthalmologist's monitoring	YES
6	Topical calcineurin inhibitors, preferentially cyclosporine A (0.1% on-label in EU), may be used as a steroid-sparing agent in steroid-dependent patients followed in specialized centers; tacrolimus 0.1% eye drops should be reserved for severe VKC and AKC cases refractory to cyclosporine (off-label in EU)	Y/N
7	Systemic immunosuppressive treatment should be prescribed in most refractory cases of AKC with visual threat. Cyclosporine is the most frequently used drug. Tacrolimus and mycophenolate mofetil are alternative options	Y/N

AKC, atopic keratoconjunctivitis; *N*, agreement < 50%; PAC, perennial allergic conjunctivitis SAC, seasonal allergic conjunctivitis VKC, vernal keratoconjunctivitis; Y/N, agreement between 50 and 75%; YES, agreement between expert > 75%.

## Acknowledgements

*None.*

### Financial support and sponsorship

*None.*

### Conflicts of interest

*There are no conflicts of interest.*

## REFERENCES AND RECOMMENDED READING

Papers of particular interest, published within the annual period of review, have been highlighted as:

▪ of special interest
▪▪ of outstanding interest
